# Impact of microRNA-130a on the neutrophil proteome

**DOI:** 10.1186/s12865-015-0134-8

**Published:** 2015-11-25

**Authors:** Corinna Cavan Pedersen, Jan Christian Refsgaard, Ole Østergaard, Lars Juhl Jensen, Niels Henrik Helweg Heegaard, Niels Borregaard, Jack Bernard Cowland

**Affiliations:** The Granulocyte Research Laboratory, Department of Hematology, National University Hospital, University of Copenhagen, 9322, Blegdamsvej 9, DK-2100 Copenhagen Ø, Denmark; Disease Systems Biology Program, Novo Nordisk Foundation Center for Protein Research, Faculty of Health and Medical Sciences, University of Copenhagen, Blegdamsvej 3B, DK-2200 Copenhagen N, Denmark; Department of Autoimmunology & Biomarkers, Statens Serum Institut, Artillerivej 5, DK-2300 Copenhagen S, Denmark; Department of Clinical Biochemistry and Pharmacology, Odense University Hospital, University of Southern Denmark, J.B. Winsløws Vej 19, DK-5000 Odense C, Denmark

**Keywords:** miR-130a, Neutrophils, pSILAC, Quantitative proteomics, RAIN, miRNA target network

## Abstract

**Background:**

MicroRNAs (miRNAs) are important for the development and function of neutrophils. miR-130a is highly expressed during early neutrophil development and regulates target proteins important for this process. miRNA targets are often identified by validating putative targets found by *in silico* prediction algorithms one at a time. However, one miRNA can have many different targets, which may vary depending on the context. Here, we investigated the effect of miR-130a on the proteome of a murine and a human myeloid cell line.

**Results:**

Using pulsed stable isotope labelling of amino acids in cell culture and mass spectrometry for protein identification and quantitation, we found 44 and 34 proteins that were significantly regulated following inhibition of miR-130a in a miR-130a-overexpressing 32Dcl3 clone and Kasumi-1 cells, respectively. The level of miR-130a inhibition correlated with the impact on protein levels. We used RAIN, a novel database for miRNA–protein and protein–protein interactions, to identify putative miR-130a targets. In the 32Dcl3 clone, putative targets were more up-regulated than the remaining quantified proteins following miR-130a inhibition, and three significantly derepressed proteins (NFYC, ISOC1, and CAT) are putative miR-130a targets with good RAIN scores. We also created a network including inferred, putative neutrophil miR-130a targets and identified the transcription factors Myb and CBF-β as putative miR-130a targets, which may regulate the primary granule proteins MPO and PRTN3 and other proteins differentially expressed following miR-130a inhibition in the 32Dcl3 clone.

**Conclusion:**

We have experimentally identified miR-130a-regulated proteins within the neutrophil proteome. Linking these to putative miR-130a targets, we provide an association network of potential direct and indirect miR-130a targets that expands our knowledge on the role of miR-130a in neutrophil development and is a valuable platform for further experimental studies.

**Electronic supplementary material:**

The online version of this article (doi:10.1186/s12865-015-0134-8) contains supplementary material, which is available to authorized users.

## Background

Neutrophils are the most abundant leukocytes in human blood. They are essential for innate immunity and are the first cells to be recruited for defence against invading microorganisms at sites of infection [1]. Neutrophils are continuously produced in the bone marrow through a tightly regulated process termed granulopoiesis. Different types of granules loaded with microbicidal proteins appear at different stages of neutrophil differentiation, which is defined by six morphologically distinguishable cellular stages of maturation. The first morphologically identified precursor committed to the neutrophil lineage is the myeloblast. Myeloblasts mature into promyelocytes, in which formation of primary granules containing myeloperoxidase (MPO) takes place, and further to myelocytes where secondary granules are formed. Proliferation ceases as differentiation proceeds via the metamyelocyte, the band cell, and ultimately the mature segmented neutrophil, which is the cell released to blood. Granulopoiesis is strictly regulated by transcription factors including RUNX1, C/EBPα, and C/EBPε [2–4].

MicroRNAs (miRNAs) are small, non-coding RNAs that negatively regulate gene expression on a post-transcriptional level by binding to target mRNAs, thereby altering translation of the transcripts or their stability. Target recognition is mediated through base-pairing of the miRNA with its target mRNA, usually between a 6–7 nucleotide seed sequence situated within residues 2–8 of the miRNA and a miRNA recognition element (MRE) in the 3′ untranslated region (3′ UTR) of the mRNA [5, 6]. The resulting changes in protein expression affect widespread cellular processes including development, differentiation, proliferation, survival, and immune responses [7, 8]. Alterations in miRNA levels have been associated with numerous diseases and are implicated in the initiation and progression of human cancers including leukemias [8].

We recently demonstrated that miRNAs are regulators of proteins essential for granulopoiesis [9–11]. We showed that miR-130a suppresses expression of Smad4 and thereby reduces sensitivity to TGF-β1-induced growth inhibition [9]. miR-130a also represses appropriate cell cycle exit and secondary granule protein expression by targeting C/EBPε in neutrophil precursors [9, 11]. These two targets for miR-130a were suggested by *in silico* prediction algorithms based on conservation across species, sequence complementarity, and other miRNA–mRNA binding properties [12, 13]. This is an often-used approach to identify putative targets of miRNAs. However, many predicted targets are false positives, and several factors that may influence the effect of the miRNA on the mRNA are not taken into consideration [14]. These include tissue specificity, expression levels of both RNA molecules, and the surrounding regulatory mechanisms particular to the cell of interest [15, 16]. More direct methods used for finding miRNA targets include identification of changes in mRNA transcript levels induced by altered miRNA expression and immunoprecipitation of miRNA–mRNA complexes [17–21]. However, these methods do not account for the actual reduction in protein levels seen in a particular cell type in response to changes in expression of an endogenous miRNA. Protein turnover times vary, and repression of gene expression by miRNAs can occur solely through translational inhibition, altering only the amount of protein being produced and not the amount of mRNA [9]. Immunoblotting is frequently used to determine the effect of miRNA-mediated reduction on synthesis of selected proteins. As an alternative for a more extensive, global characterization of the effects of miRNA modulations on protein expression, stable isotope labelling of amino acids in cell culture (SILAC) and mass spectrometry (MS) may be used as originally shown [22] and applied to the neutrophil proteome in a murine model [23]. Further developments of the approach include pulsed SILAC (pSILAC), in which only newly synthesized proteins are labelled as shown in the analysis of around 5000 proteins in HeLa cells [18].

Here, we used pSILAC-based MS to investigate the effects of miR-130a on the neutrophil proteome using two myeloblast-derived cell lines: murine 32Dcl3 cells and human Kasumi-1 cells. miR-130a is highly expressed in early neutrophil precursors (myeloblasts and promyelocytes) compared to more mature precursors [9, 10]. We demonstrate that inhibition of miR-130a with an anti-miR-130a oligonucleotide in a miR-130a-overexpressing 32Dcl3 clone and in Kasumi-1 cells results in significant changes in the levels of 44 out of 2092 proteins and 34 out of 1238 proteins, respectively. We found that putative miR-130a target proteins are more induced than the remaining proteins quantified in the 32Dcl3 miR-130a clone following miR-130a inhibition. Three significantly induced proteins, nuclear transcription factor Y, gamma (NFYC), isochorismatase domain containing 1 (ISOC1), and catalase (CAT), are putative miR-130a targets with good scores in RAIN (RNA–protein Association and Interaction Networks) [http://rth.dk/resources/rain/]. Finally, we constructed miR-130a target protein networks including the proteins identified in this study in order to find pathways affected by miR-130a in the context of neutrophil development. We identified important myeloid regulatory proteins, such as Myb and Core-binding factor beta (CBF-β), as putative direct miR-130a targets.

## Methods

### Cell culture, transfection, and pSILAC labelling

The sequences of murine and human miR-130a-3p (miR-130a) are identical. The murine myeloblast-derived cell line 32Dcl3 (ATCC® CRL-11346) was stably transfected with the expression plasmid pEGP-miR-130a (Cell Biolabs) as described previously [9]. This 32Dcl3 miR-130a clone was cultured in SILAC medium consisting of RPMI medium without arginine, lysine, and glutamine (PAA Cell Culture Company), 10 % dialysed FBS (Gibco), 100 U/mL penicillin and 100 μg/mL streptomycin (Gibco), 1 ng/mL murine IL-3 (Sigma-Aldrich), 1 % GlutaMAX™-1 (Gibco), and 0.2 mg/mL proline (Sigma-Aldrich) to avoid arginine-to-proline conversion [24]. Either naturally occurring isotopes of lysine and arginine (light (L)), 4,4,5,5-D_4_-lysine and ^13^C_6_-arginine (Lys4 & Arg6, medium-heavy (M)), or ^13^C_6_,^15^N_2_-lysine and ^13^C_6_,^15^N_4_-arginine (Lys8 & Arg10, heavy (H)) were added to the medium at a concentration of 48.67 mg/L for lysine and 28 mg/L for arginine.

For pSILAC, the 32Dcl3 miR-130a clone was grown in SILAC L medium for six days and subsequently washed twice with phosphate-buffered saline (PBS) to eliminate traces of L amino acids. Cells (5x10^6^/condition) were then transfected with anti–miR-130a-LNA or scrambled-LNA (Exiqon) through electroporation using the AMAXA nucleofection system (program E-032) according to the manufacturer’s recommendations and transferred to M (anti–miR-130a-LNA) or H (scrambled-LNA) medium for approximately 48 h. Similarly, Kasumi-1 cells (human myeloblast cell line derived from a patient with acute myeloblastic leukemia, ATCC® CRL-2724) were grown in normal medium (RPMI1640 (Gibco), 20 % FBS (Gibco), 100 U/mL penicillin and 100 μg/mL streptomycin (Gibco)), washed twice with PBS before transfection (program C-23) with anti–miR-130a-LNA or scrambled-LNA (Exiqon) and switched to M or H SILAC medium, respectively, for approximately 72 h. The SILAC medium used for Kasumi-1 cells was identical to the 32Dcl3 SILAC medium except 20 % dialysed FBS was used and no IL-3 was added.

### RNA and protein extraction and quantification

Kasumi-1 cells were centrifuged on a Histopaque 1.077 gradient (400 g, 4 °C, 30 min) to remove dead cells. Both cell lines were washed twice in PBS and counted with an automatic cell counter (Coulter Z1; Beckman). Viability was evaluated using trypan blue (Sigma-Aldrich) exclusion. Total RNA was extracted from cells from each condition with the PureLink RNA Mini Kit (Ambion) according to the manufacturer’s recommendations. M and H conditions were combined 1:1 based on cell counts. For protein extraction, cell combinations were lysed in preheated, non-reducing 2x Laemmli sample buffer (125 mM Tris–HCl pH 6.8, 4 % SDS, 20 % glycerol) supplemented with cOmplete Mini Protease Inhibitor Cocktail (Roche) and boiled for 10 min. Protein concentration was measured using the Pierce BCA Protein Assay kit (Thermo Scientific).

### cDNA synthesis and miRNA detection

Reverse transcription of miRNA to first-strand cDNA was performed using the primers miR-130a (RT454) with SNO234 (RT1234, Applied Biosystems) and RNU6B (RT1093, Applied Biosystems) as internal normalizers in 32Dcl3 and Kasumi-1 cells, respectively. Real-time PCR was performed in triplicate using TaqMan MicroRNA Assays (20x) (all Applied Biosystems) for miR-130a (RT454) with SNO234 (RT1234) and RNU6B (RT1093) as controls on a 3000-P real-time PCR machine (Stratagene).

### In-solution digestion

From the cell lysate, 50 μg of protein were precipitated using trichloroacetic acid/acetone followed by protein re-solubilization in 10 μL 8 M urea, 50 mM NH_4_HCO_3_. The re-solubilized protein was subsequently reduced by incubation with dithiothreitol (10 mM final concentration, 30 min, room temperature) followed by alkylation with iodoacetamide (50 mM final concentration, 30 min in the dark at room temperature). The alkylated proteins were next pre-digested for three hours using endo-Lys C (final concentration 0.5 μg endo-Lys C/50 μg protein; Waco Pure Chemical Industries) before dilution to a final concentration of 2 M urea and continued digestion overnight at room temperature using trypsin (final concentration 1 μg trypsin/50 μg protein, Promega). Peptides were then depleted for SDS using Pierce Detergent Removal Spin Columns (Thermo Scientific, Rockland, IL, USA) before OFFGEL peptide fractionation.

### OFFGEL peptide fractionation

Tryptic peptides were fractionated according to their isoelectric points into 12 fractions by OFFGEL fractionation on a 13 cm immobilized pH gradient strip (pH 3-10NL, GE Healthcare) using a 3100 OFFGEL separator (Agilent Technologies). Fractions were collected into Eppendorf tubes and dried down to a final volume of 20 μL using a vacuum concentrator (Eppendorf) before freezing or further processing.

### Peptide desalting using StageTips

Concentrated OFFGEL fractions were diluted with 5 % formic acid and desalted on a pre-equilibrated homemade StageTip essentially as described previously [25]. The desalted peptides were then incubated in a vacuum concentrator (Eppendorf) to almost complete dryness for methanol removal and then re-dissolved into 20 μL 5 % formic acid and transferred to a glass vial for further analysis by MS.

### Peptide analysis by LC-MS/MS

Five microliters of the desalted OFFGEL peptide fractions were loaded on an Acclaim PepMap100 C18 precolumn (100 μm x 2 cm, 5 μm particle size, 100 Å, Thermo Fischer Scientific). Proteins were separated using an EASY-spray PepMap100 C18 analytical column with integrated emitter (75 μm inner diameter, 150 mm long, 3 μm particle size, 100 Å, Thermo Fisher Scientific) on a 90-min gradient controlled by an Easy-nLC II (Thermo Fisher Scientific). The column was connected to an LTQ Orbitrap XL mass spectrometer (Thermo Fisher Scientific) equipped with an EASY-spray nano-electrospray source (Proxeon, Odense, Denmark). The flow rate was 200 nL/minute; the mobile phases consisted of solvent A (2 v/v % acetonitrile, 0.1 % formic acid) and solvent B (95 v/v % acetonitrile, 0.1 % formic acid v/v). The gradient went from 0 % to 45 % solvent B in 80 min, followed by 10 min with 100 % solvent B; then data acquisition was stopped and the column was re-equilibrated with solvent A. MS data were acquired recording full scan spectra (300–1800 mass/charge [m/z]) in the Orbitrap with 60,000 resolution at 400 m/z. MS/MS data were recorded in parallel in a data-dependent mode, fragmenting the 5 most abundant ions (charge state +2 or higher) by collision-induced dissociation in the LTQ ion trap at 35 % collision energy. MS/MS spectra were recorded using dynamic exclusion (30 s) to minimize repeated fragmentation of the same peptides.

### Data analysis using MaxQuant

Recorded raw files were analysed using MaxQuant version 1.1.1.36 [26] for peptide quantitation by MS1-intensity and for protein identification using the Andromeda search engine [27] with the following settings: FASTA-files: Homo_sapiens.GRCh37.73.pep.all.fasta (Kasumi-1 cells) or Mus_musculus.GRCm38.73.pep.all.fasta (32Dcl3 cells). Variable modifications: oxidation (M), acetyl (protein *N*-terminal), fixed modifications: carbamidomethyl (C). Labels: Arg6 & Lys4 as medium-labelled and Arg10 & Lys8 as heavy-labelled amino acids. Peptide false discovery rate (FDR): 1 %. Protein FDR: 1 %. Match between runs: 2 min. Keep low-scoring version of identified peptides: on. All other settings were left at their defaults.

### Data analysis of proteins identified by LC-MS/MS

Significantly regulated proteins were determined by performing a Significance B test [26] with a window size of 900 and step size of 300 proteins (for more information see Additional file 1: Supplemental methods and Additional file 2: Figure S1). Assigned p-values were corrected for multiple testing using the Benjamini–Hochberg procedure [28]. Proteins with a resulting corrected p-value (q) <0.01 were deemed significantly regulated. Each protein was assigned a human readable HGNC (HUGO Gene Nomenclature Committee) or MGI (Mouse Genome Informatics) symbol via Ensembl BioMart version 0.7 [29].

Each protein was assigned a miR-130a association probability by mapping to RAIN [http://rth.dk/resources/rain/] via the STRING 10 alias file. For miRNA target identification, RAIN incorporates experimentally supported interactions (miRTarBase [30], NPInter [31], StarBase [32]), miRNA and mRNA co-occurrence-based text mining of Medline abstracts [33], as well as processed miRNA target predictions from the prediction algorithms TargetScan [34], PicTar [35], miRanda [12], and StarMirDB [36]. Based on these inputs, the program assigns association probabilities to potential targets of a given miRNA according to the combined level of confidence of the miRNA–mRNA interaction. RAIN obtains all protein–protein interactions from STRING [37].

The proteins identified in MS were compared with the top 20 % best scoring RAIN association probabilities for miR-130a to a) find potential miR-130a targets among the quantified proteins for the two cell lines, and to b) identify potential miR-130a target proteins for the miR-130a association network that might associate with proteins within the regulated protein subset found for the 32Dcl3 miR-130a clone. Cytoscape [38] was used to visualize this network. The DAVID tool [39, 40] was used to identify enriched Gene Ontology terms for cellular components, and the Ingenuity Pathway Analysis software (QIAGEN, http://www.qiagen.com/ingenuity) was used to identify enriched ‘Molecular and Cellular Functions’ among regulated proteins compared to the remaining quantified proteins within the same experiment.

## Results and discussion

### miR-130a expression in the 32Dcl3 and Kasumi-1 cell lines

Our aim was to evaluate the effect of miR-130a on the proteome at the time of neutrophil maturation when miR-130a expression is at its peak (myeloblasts and promyelocytes) [9]. We therefore chose a murine and a human cell line both representing immature neutrophil precursors. The murine myeloblast-derived cell line 32Dcl3 was stably transfected with a miR-130a-expressing plasmid, resulting in a clone over-expressing miR-130a. This has previously been shown to be a good model for investigating the effect of miR-130a on specific target proteins [9, 11]. The Kasumi-1 cell line is derived from an acute myeloid leukemia patient with t(8;21)(q22;q22) chromosomal translocation and has a 2.5-fold higher endogenous expression of miR-130a than the level found in primary myeloblasts and promyelocytes isolated from normal human bone marrow [9].

### pSILAC approach for identification of myeloid miR-130a target proteins

**Fig. 1:**
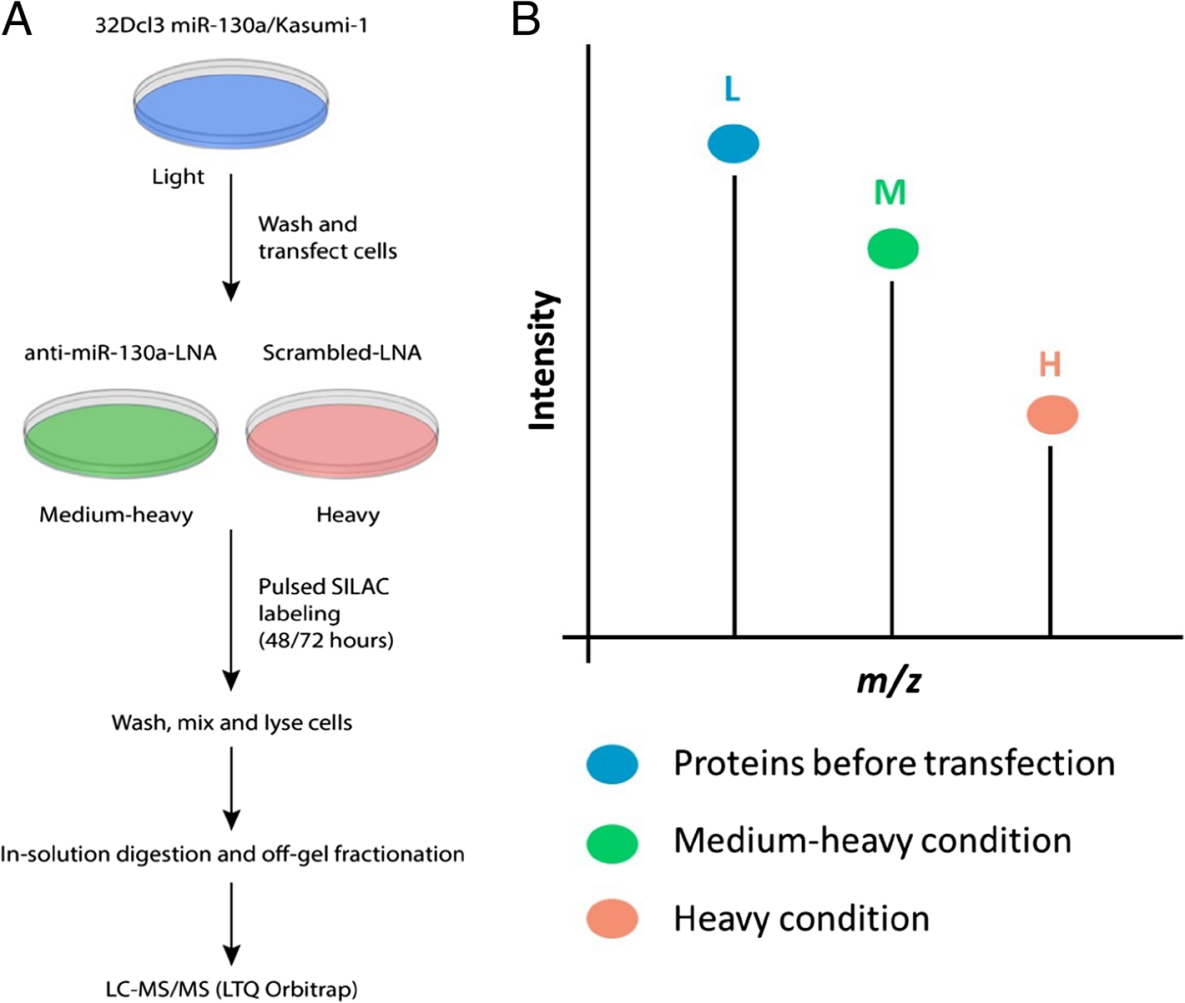
Experimental setup of the pSILAC method. **a** Cells grown in L medium were transfected with anti-miR-130a-LNA or scrambled-LNA (mock-transfection) and transferred to M or H SILAC-medium, respectively. After 48 h (32Dcl3 miR-130a clone, doubling time ~18–20 h) or 72 h (Kasumi-1, doubling time ~40 h) of pulse labelling, cells were washed, combined 1:1, and lysed. Samples were prepared for and analysed by LC-MS/MS, producing peptide peaks as shown (**b**). The light peptides were disregarded while the M and H peptides were compared, generating M/H ratios for further analysis

We transiently transfected the 32Dcl3 miR-130a clone and Kasumi-1 cells with an inhibitory LNA probe against miR-130a (anti-miR-130a-LNA) or a scrambled-LNA (mock transfection). The cells were then transferred to medium-heavy (M) and heavy (H) medium, respectively, and pulsed for approximately 48 h (32Dcl3) or 72 h (Kasumi-1). At this point, the effect of miR-130a is reflected by differences between M and H proteins while light (L) proteins have all been synthesized prior to interference with the free miR-130a pool. Subsequently, cells from each condition were combined in equal amounts, lysed, and prepared for analysis on the LTQ Orbitrap XL mass spectrometer after extensive fractionation as described in Methods. The experimental setup is outlined in Fig. 1. The free pool of miR-130a at the time of harvest was measured using real-time PCR and indicates the different levels in the anti-miR-130a-LNA and scrambled-LNA conditions (Additional file 3: Figure S2).

### Impact of miR-130a on protein output

**Fig. 2:**
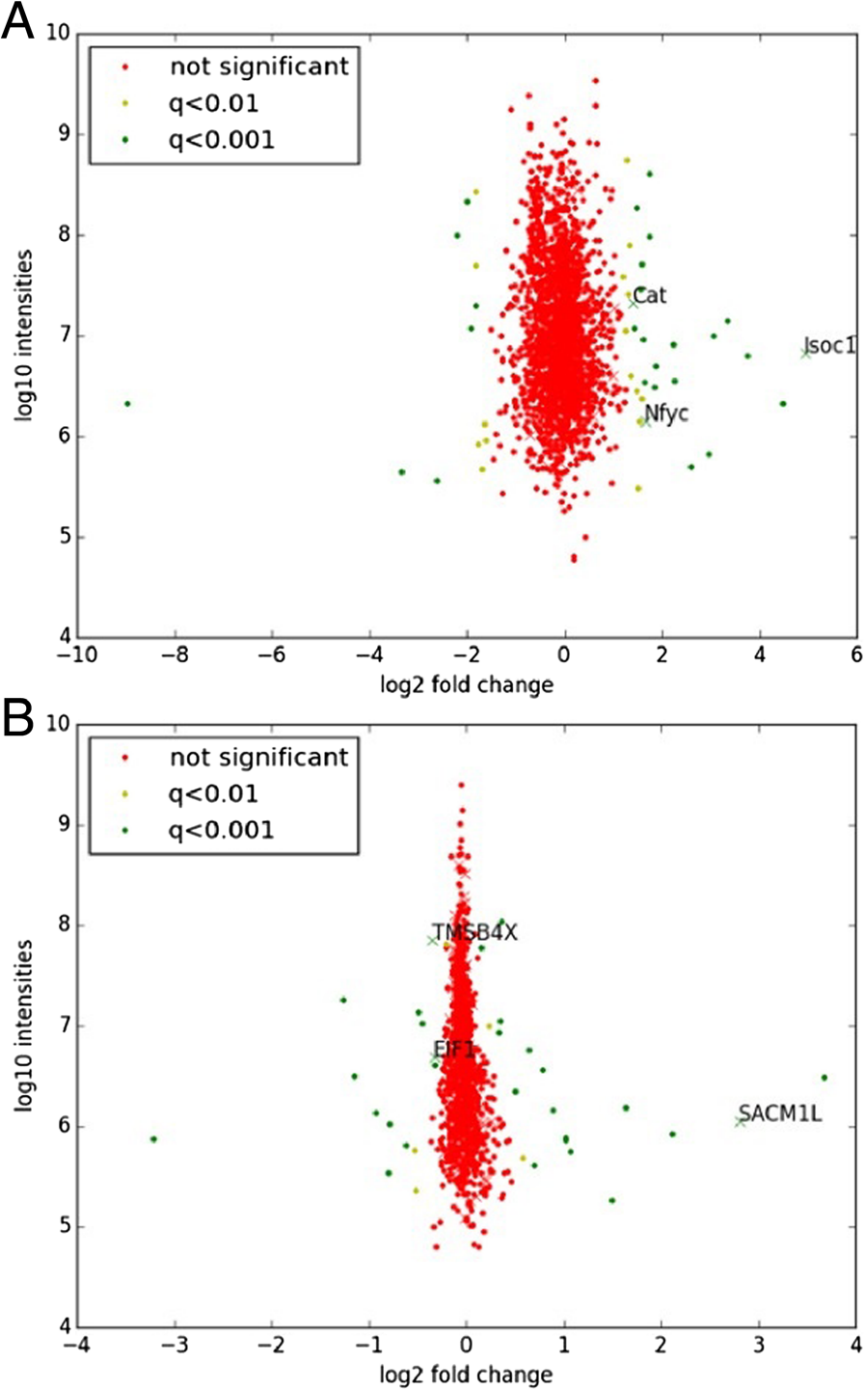
The intensities in MS as a function of log2 fold changes in protein expression between the anti–miR-130a-LNA and scrambled-LNA conditions for the 32Dcl3 miR-130a clone (**a**) and the Kasumi-1 cells (**b**). Proteins are represented by crosses (miR-130a association probability in top 20 %) or dots (miR-130a association probability below top 20 % or no score). Colours represent significance according to the Significance B test (Benjamini-Hochberg corrected). Significantly regulated proteins with q < 0.01 and top 20 % miR-130a association probabilities are given by name

For the 32Dcl3 miR-130a clone, we identified a total of 2092 proteins present both in the M and H condition and their ratios could be quantified. For the Kasumi-1 cells, this number was 1238 proteins, possibly reflecting the slower growth and incorporation of new amino acids in these cells. Additional file 4 lists the complete sets of quantified proteins and related parameters, and Fig. 2 shows the intensities in MS as a function of log2 fold changes in protein expression. Proteins differentially regulated by miR-130a were determined based on statistical significance rather than a fold change cut-off. In this manner, differences in noise at different levels of intensities within the MS analysis were taken into consideration. Moreover, the effect of miRNAs can be rather subtle [18] and might not amount to *e.g.* a two-fold change in target protein levels. After correction for multiple testing, we identified 31 up-regulated and 13 down-regulated proteins in the 32Dcl3 miR-130a clone, and 19 up-regulated and 15 down-regulated proteins in Kasumi-1 cells, following miR-130a inhibition (q < 0.01).

### Biological characteristics of neutrophil miR-130a-regulated proteins

We investigated the biological characteristics of the regulated protein subset in the 32Dcl3 miR-130a clone. Lysosomes and peroxisomes were found by DAVID to be enriched cellular components in the regulated subset compared to all quantified proteins. Using the Ingenuity Pathway Analysis software, we also found that ‘cellular development’ and ‘cellular growth and proliferation’ were the two most enriched ‘Molecular and Cellular Functions’ in the regulated subset compared to the remaining quantified proteins. This corresponds well with observed effects of miR-130a on proliferation and cell cycle in immature neutrophil cells [9, 11].

### Identification of miR-130a targets in the neutrophil proteome

The standard way to validate the impact of a miRNA in a proteomics study is to search for an enrichment of matches to the miRNA seed sequence within the 3′ UTRs of the mRNAs encoding proteins found to be regulated within the experiment [41, 42]. However, several other aspects of the miRNAs and of their target mRNAs are involved in their interaction, including mRNA accessibility and complementarity between the remaining miRNA sequence and the mRNA target [43]. Different *in silico* miRNA target prediction algorithms take these and other aspects into consideration to various extents, resulting in algorithms with distinct specificity and sensitivity and, consequently, varying reliability in their identification of potential miRNA targets [44]. While the seed sequence is commonly accepted as being an indispensable part of the interaction mechanism [6], its presence alone grossly overestimates the number of targets for a given miRNA [44]. We therefore used a novel tool, RAIN [http://rth.dk/resources/rain/], to identify potential direct miR-130a targets among the proteins found within our two studies (see Methods for details). As most of the associations come from prediction algorithms, we chose the top 20 % highest scoring associations, which consist of 678 predictions, 5 experiments, and 6 text-mining interactions for miR-130a in mice.

**Fig. 3:**
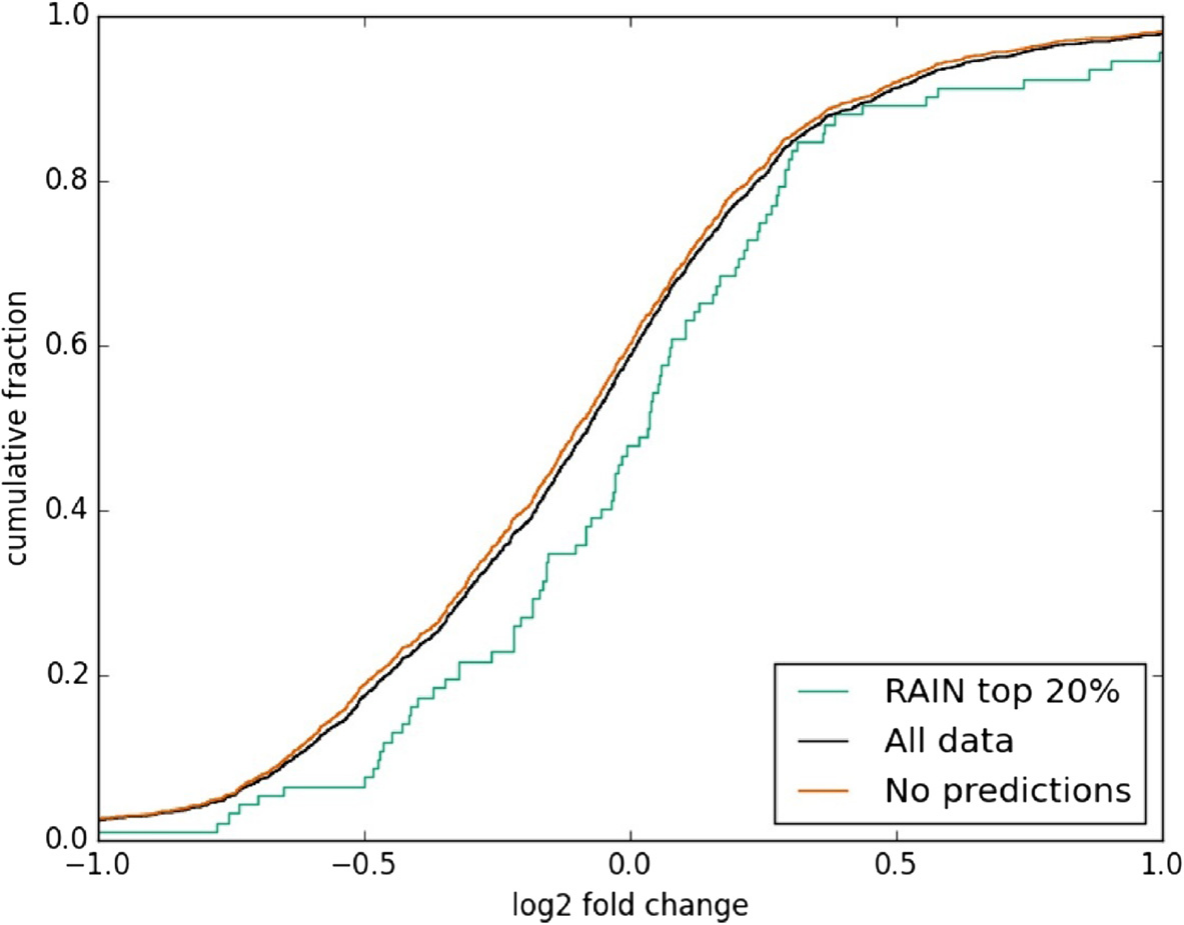
The cumulative distributions of M/H ratios of proteins with good RAIN scores (top 20 %, green), all remaining proteins (black), and proteins without RAIN scores (orange) as a function of log2 M/H fold changes in protein expression between the anti-miR-130a-LNA (M) and scrambled-LNA (H) conditions for the 32Dcl3 miR-130a clone. A Kolmogorov-Smirnov test of equality between distributions results in the following: *p*-value = 0.018 for the comparison between M/H ratios of proteins with good RAIN scores and the M/H ratios of proteins without scores; *p*-value = 0.042 for the M/H ratios of proteins with good RAIN scores and the M/H ratios of all remaining proteins

First, we used RAIN to determine whether changes in protein levels could be directly attributed to the inhibition of miR-130a. We compared the fold changes of potential miR-130a target proteins with a good RAIN score (top 20 %) to those of proteins without scores (Fig. 3). We found potential targets to be more up-regulated after miR-130a inhibition for the 32Dcl3 miR-130a clone (Kolmogorov–Smirnov test, *p*-value = 0.018) as demonstrated by the right shift of the curve showing higher M/H ratios. The effect was not present for the Kasumi-1 cells (not shown). This confirms that there is an overall greater effect of inhibiting miR-130a in cells from the 32Dcl3 miR-130a clone where the free miR-130a pool is much more reduced (~31 fold reduction) than in the Kasumi-1 cell line (~2.3 fold reduction) upon LNA-mediated inhibition (Additional file 3: Figure S2).

Secondly, we used RAIN to determine potential miR-130a targets within the subsets of significantly regulated proteins. Three derepressed proteins (NFYC, ISOC1, and CAT) with miR-130a association probabilities within the top 20 % were found in the 32Dcl3 miR-130a clone and one (Phosphatidylinositide phosphatase SAC1, SACM1L) in Kasumi-1 cells (Fig. 2), indicating that most of the de-regulated proteins are either indirectly regulated by miR-130a or *bona fide* miR-130a targets not found by RAIN.

In MS experiments, such as the one described here, typically only a minor fraction of the proteins present in the cell is detected [45]. The number is further limited by the requirement for detection of both M and H proteins to identify ratios between conditions. This may also explain the fact that the subsets of significantly regulated proteins did not overlap between the two cell lines. Proteins in the regulated subset in one cell line identified for both the H and M condition among non-significantly regulated proteins in the other cell line are listed in Additional file 5: Table S1.

In MS the capacity to detect proteins of low abundance, such as transcription factors and other regulatory proteins, is limited in samples with a high dynamic range [45–47]. In contrast, miRNAs favour transcription factors, which are generally expressed at relatively low levels in the cell [47], as targets [33]. C/EBPε is not expressed in 32Dcl3 or Kasumi-1 cells (data not shown), and Smad4 was not identified in either cell line. Consequently, a small change in the protein level of, for example, a transcription factor may have a greater and thus detectable downstream effect. Together RUNX1 and CBF-β constitute an important heterodimeric, myeloid transcription factor. CBF-β is a highly predicted miR-130a target (top 20 %). CBF-β is up-regulated 1.35-fold (although not statistically significant) in the 32Dcl3 miR-130a clone following miR-130a inhibition and a candidate upstream regulator of three proteins within the regulated subset: MPO, IL-2Ra (Ingenuity Pathway analysis) and proteinase 3 (PRTN3, author observations, unpublished). Of these three only MPO and PRTN3 were identified in the Kasumi-1 experiment, and only PRTN3 was borderline significantly up-regulated (q = 0.0113), probably reflecting the greater degree of miR-130a inhibition by anti-miR-130a-LNA observed in the 32Dcl3 miR-130a clone (Additional file 3: Figure S2).

**Fig. 4:**
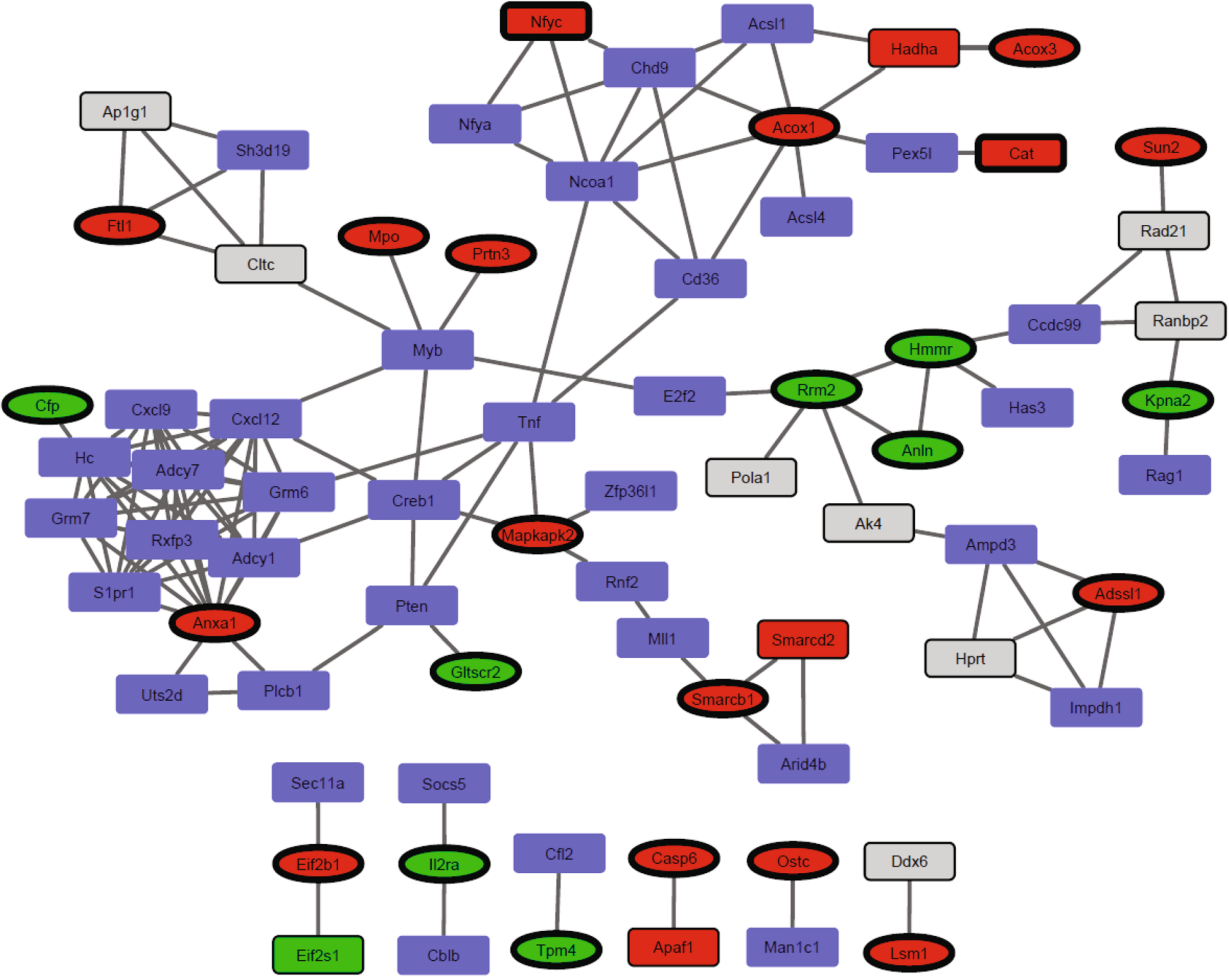
RAIN networks of potential miR-130a targets (with miR-130a association probabilities in the top 20 %) and differentially regulated proteins identified for the 32Dcl3 miR-130a clone following miR-130a inhibition. Only proteins with STRING association probabilities above 0.7 and interactions with significantly regulated proteins are included. Square: predicted target (= top 20 %). Oval: not predicted. Bold outline: in dataset and significant. Thin outline: in dataset but not significant. No outline and blue: not in dataset. Red: up-regulated >0.3 (log2 ratio) upon miR-130a inhibition. Green: down-regulated < −0.3 (log2 ratio) upon miR-130a inhibition. Grey: regulated less than 0.3 (log2 ratio). These fold changes are included in the network to also indicate the observed direction of strongly predicted targets quantified but not significantly regulated within the data set

To identify additional potential regulatory networks involving miR-130a, we constructed association networks for the subset of 44 regulated proteins found for the 32Dcl3 miR-130a clone. Other proteins that interact with this protein subset with protein-protein association probabilities above 0.7 and which have miR-130a association probabilities within the top 20 % were also included. This resulted in a major miR-130a network as well as several minor, disconnected networks (Fig. 4). The major miR-130a network includes the transcriptional activator Myb, which is more highly expressed in early neutrophil precursors compared to more mature cells [48]. Myb is a predicted miR-130a target but was not identified in either cell line. If Myb is up-regulated following inhibition of miR-130a, this could lead to the observed increases of its targets, the primary granule proteins MPO [49] and PRTN3 [50] (Fig. 4). Therefore, both CBF-β and Myb are potential direct miR-130a targets worth investigating further. CBF-β, Myb, and miR-130a are co-expressed in early myeloid precursors, suggesting that miR-130a may have a buffer effect on their protein levels ([48, 51] and unpublished data).

In murine cells, RAIN does not identify Smad4 as a miR-130a target. TargetScan identifies Smad4 as a human miR-130a target with two seed matches (MREs) in the *Smad4* 3′ UTR. The same MREs are also found in the murine *Smad4* 3′ UTR, but presumably other factors of the TargetScan algorithm prevent its prediction of murine Smad4 as a miR-130a target since murine and human miR-130a are identical. We have previously experimentally identified Smad4 as a direct miR-130a target in murine cells [9]. However, Medline abstracts and experimental databases in RAIN only include murine targets if it is explicitly stated that they are derived from experiments with murine cells. Therefore, it cannot be excluded that certain interesting miR-130a targets will be missed using RAIN due to this restriction in the underlying databases. RAIN does identify a weak interaction, although below the network cut-off, between Smad4 and NFYC, which is significantly up-regulated upon miR-130a inhibition. NFYC is a predicted miR-130a target, but the effect of miR-130a might also be mediated by Smad4 or one of its other associated network proteins such as its heterotrimeric transcription factor complex partner NFYA. This complex plays an important role in cell cycle progression [52] and may thus be regulated in neutrophil precursors by miR-130a targeting two of its subunits.

Not much is known about ISOC1, also significantly derepressed by miR-130a inhibition and a highly predicted miR-130a target, other than that it associates with peroxisomes, has enzymatic activity, and may promote cell growth [53, 54]. The last miR-130a target with a good RAIN score and significantly derepressed protein in the 32Dcl3 miR-130a clone is CAT. CAT is also usually located within peroxisomes and catalyses hydrogen peroxide degradation [55]. The significantly down-regulated Smarcb1 associates with Smarcd2, which is a predicted miR-130a target and down-regulated, although not significantly, in this study. These proteins form parts of SWI/SNF complexes, which regulate gene expression through chromatin remodelling [56]. This indicates that, in addition to transcription factors, miR-130a may target other types of transcriptional regulators as well as metabolic processes in neutrophil precursors. In summary, these and other interactions identified here can form the basis for further experimental identification of miR-130a regulation in neutrophil development extending further than just single miR-130a–mRNA interactions.

## Conclusion

We identified subsets of the murine and human neutrophil proteomes significantly regulated by miR-130a, which likely represent a mixture of direct targets, including NFYC, ISOC1, and CAT, and mainly indirect miR-130a targets. We demonstrated that substantial inhibition of miR-130a affects the overall expression of predicted target proteins in the murine neutrophil model system. We identified common biological features of the significantly regulated murine protein subset. Combining the significantly regulated murine protein subset with high-scoring putative miR-130a targets from the RAIN database in an interaction network, we identified subsets of proteins with potential roles in downstream miR-130a regulation relevant for further experimental investigation. Based on these analyses, we identified Myb and CBF-β as putative direct miR-130a targets and potential regulators of the primary granule proteins MPO and PRTN3 following miR-130a inhibition in the 32Dcl3 miR-130a clone. Together, these results provide significant insight into multiple miR-130a-regulated proteins and emphasize its important regulatory role in neutrophil development.

## Additional files

Additional file 1:
**Supplemental methods.** Assignment of p-values to ratios. (DOC 23 kb) Additional file 2: Figure S1. Assignment of p-values to ratios (shown for the 32Dcl3 miR-130a clone). The top row shows the bins (approximately size 300), the other rows show which bins were used to estimate the standard deviation. For instance, the standard deviation of ratios in bin 3 was estimated based on window 3 which contained bin 2, 3 and 4. (TIF 63 kb) Additional file 3: Figure S2. Expression of miR-130a following transfection with an anti-miR-130a-LNA or scrambled-LNA of the 32Dcl3 miR-130a clone for 48 h (left) and Kasumi-1 cells for 72 h (right) measured by real-time PCR. The large difference in the level of free miR-130a in the 32Dcl3 miR-130a clone compared to Kasumi-1 cells is presumably due to the former cells being more susceptible to transfection than the latter. Error bars represent SD between triplicate measurements in the real-time PCR experiment. (TIF 44 kb) Additional file 4:
**Complete sets of quantified proteins and related parameters in the 32Dcl3 miR-130a clone and the Kasumi-1 cells.** (XLSX 1936 kb) Additional file 5: Table S1. A. H/M ratios of significantly regulated proteins in the 32Dcl3 miR-130a clone also identified and quantified for the Kasumi-1 cell line. B. H/M ratios of significantly regulated proteins in the Kasumi-1 cell line also identified and quantified for the 32Dcl3 miR-130a clone. Italic: different directions of regulation for the two cell lines. (EPS 1182 kb)

## Abbreviations

3′ UTR: 3′ untranslated region
Arg6: ^13^C_6_ Arginine
Arg10: ^13^C_6_,^15^N_4_-Arginine
CAT: Catalase
CBF-β: Core-binding factor beta
H: Heavy
FDR: False discovery rate
ISOC1: Isochorismatase domain containing 1
Lys4: 4,4,5,5-D_4_-Lysine
Lys8: ^13^C_6_,^15^N_2_-Lysine
miRNA: microRNA
M: Medium-heavy
MPO: Myeloperoxidase
MRE: miRNA recognition element
MS: Mass spectrometry
NFYA: Nuclear transcription factor Y, alpha
NFYC: Nuclear transcription factor Y, gamma
PBS: Phosphate-buffered saline
PRTN3: Proteinase 3
pSILAC: Pulsed stable isotope labelling of amino acids in cell culture
RAIN: RNA–protein Association and Interaction Networks
SACM1L: Phosphatidylinositide phosphatase SAC1
SILAC: Stable isotope labelling of amino acids in cell culture
