# Supplementary material for: Impact of microRNA-130a on the neutrophil proteome
Source: BMC Immunol. 2015 Nov 25;16:70. doi: 10.1186/s12865-015-0134-8 (PMC4659159; doi:10.1186/s12865-015-0134-8)
Supplement: Additional file 1: — Supplemental methods. Assignment of p-values to ratios. (DOC 23 kb) [file 12865_2015_134_MOESM1_ESM.doc]

# Supplemental methods

## Assignment of p-values to ratios

The Significance B test is a variation of the Significance A test. In MS data, the magnitude of the standard deviation is inversely proportional to the logarithm of the intensity. In the Significance B test, the data is therefore binned into sizes of 300 proteins according to intensity. Using this bin size, however, there is an implicit assumption that protein numbers 601 to 900 have the same level of error as they are all in the third bin. On the contrary, proteins 900 and 901 are assumed to have different error levels because they are in the third and fourth bin, respectively. To mitigate this, we created bin sizes of approximately 300 proteins, and for all bins except the first and last bin, we calculated significances using the two adjacent bins (See Additional file 2: Figure S1). This partially mitigates the problem since the estimated error for proteins 900 and 901 will now be based on 600 shared and 300 different proteins. Of note, the bin sizes used were slightly higher than 300 because 2092 is not divisible by 300. Consequently, 4 bins of size 349 and 2 bins of size 348 were created for the 32Dcl3 miR-130a clone experiment. For the Kasumi-1 experiment, the 4 bins were of size 309 or 310.
