# Supplementary figures and images for: Impact of microRNA-130a on the neutrophil proteome
Source: BMC Immunol. 2015 Nov 25;16:70. doi: 10.1186/s12865-015-0134-8 (PMC4659159; doi:10.1186/s12865-015-0134-8)

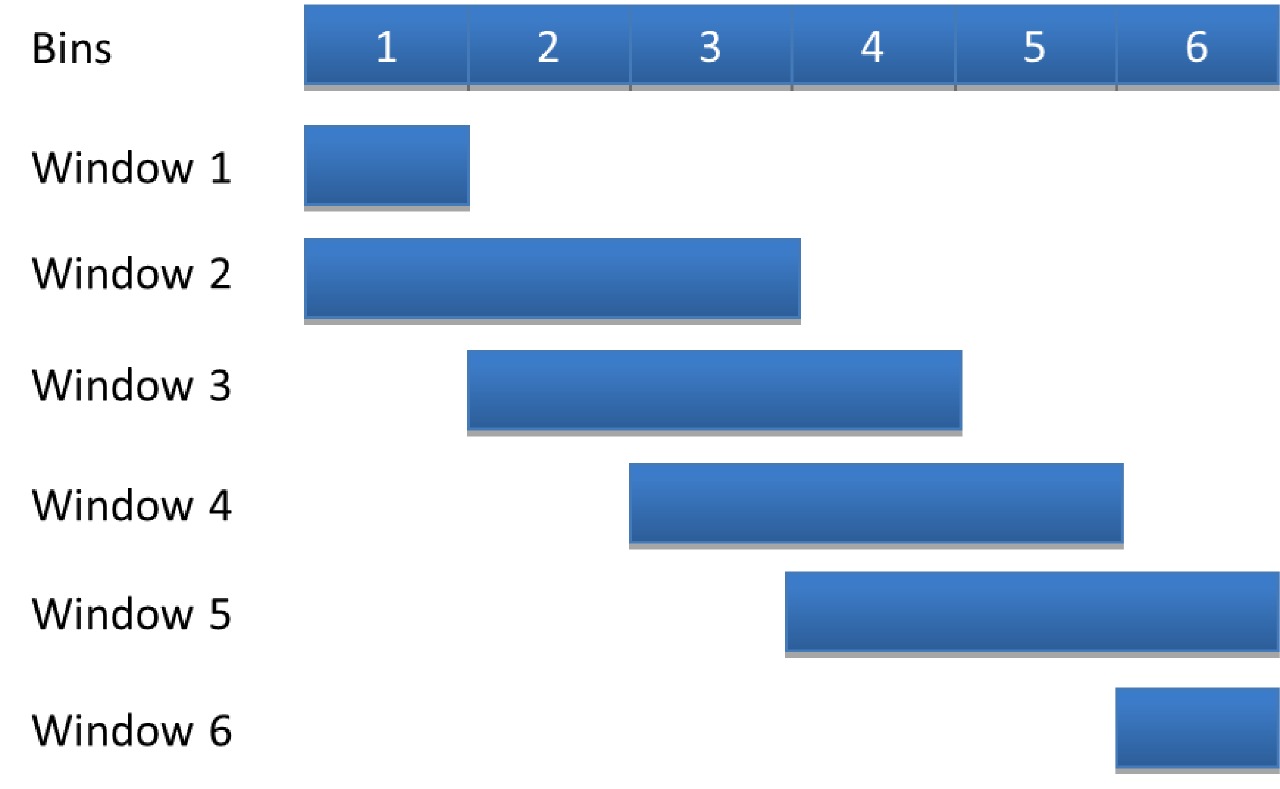

Supplement: Additional file 2: Figure S1. — Assignment of p-values to ratios (shown for the 32Dcl3 miR-130a clone). The top row shows the bins (approximately size 300), the other rows show which bins were used to estimate the standard deviation. For instance, the standard deviation of ratios in bin 3 was estimated based on window 3 which contained bin 2, 3 and 4. (TIF 63 kb) [file 12865_2015_134_MOESM2_ESM.tif]

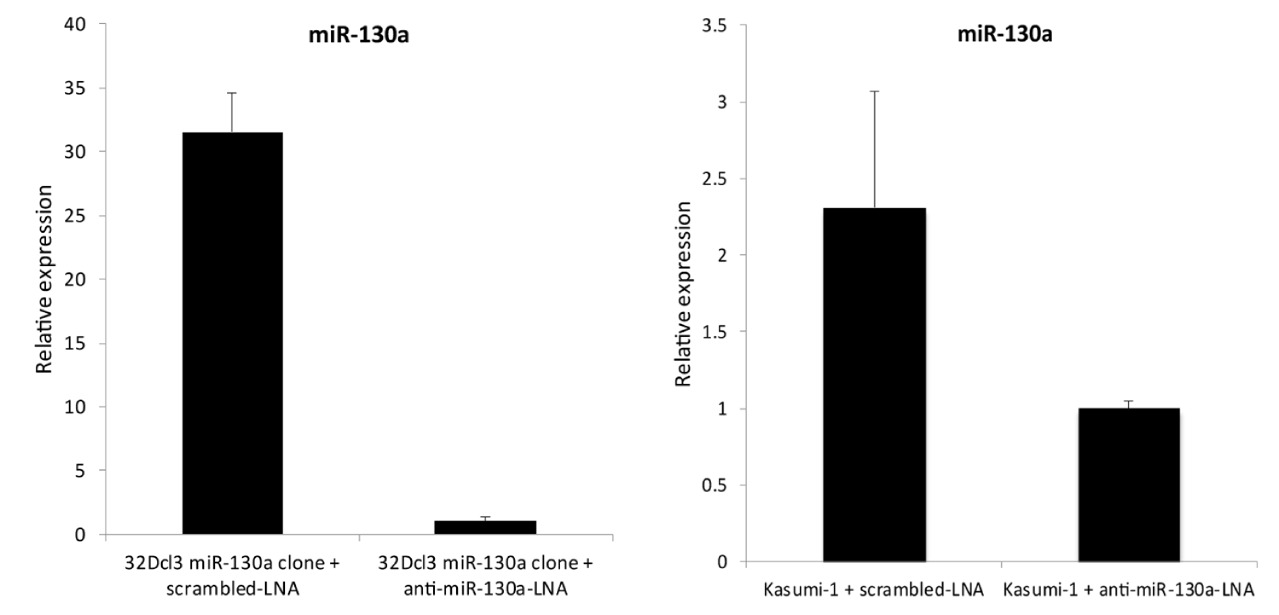

Supplement: Additional file 3: Figure S2. — Expression of miR-130a following transfection with an anti-miR-130a-LNA or scrambled-LNA of the 32Dcl3 miR-130a clone for 48 h (left) and Kasumi-1 cells for 72 h (right) measured by real-time PCR. The large difference in the level of free miR-130a in the 32Dcl3 miR-130a clone compared to Kasumi-1 cells is presumably due to the former cells being more susceptible to transfection than the latter. Error bars represent SD between triplicate measurements in the real-time PCR experiment. (TIF 44 kb) [file 12865_2015_134_MOESM3_ESM.tif]
